# Progress of Implementation of World Health Organization Global Antimicrobial Resistance Surveillance System Recommendations on Priority Pathogen-Antibiotic Sensitivity Testing in Africa: Protocol for a Scoping Review

**DOI:** 10.2196/58140

**Published:** 2024-11-15

**Authors:** Mackline Hope, Reuben Kiggundu, Dathan M Byonanebye, Jonathan Mayito, Dickson Tabajjwa, Fahad Lwigale, Conrad Tumwine, Herman Mwanja, Andrew Kambugu, Francis Kakooza

**Affiliations:** 1 Infectious Diseases Institute Kampala Uganda

**Keywords:** antimicrobial resistance, antibiotic sensitivity testing, global antimicrobial resistance surveillance system, GLASS implementation, AMR Surveillance, Africa

## Abstract

**Background:**

Antimicrobial resistance (AMR) is a major global public health concern, particularly in low- and middle-income countries where resources and infrastructure for an adequate response are limited. The World Health Organization (WHO) Global Antimicrobial Resistance Surveillance System (GLASS) was introduced in 2016 to address these challenges, outlining recommendations for priority pathogen-antibiotic combinations. Despite this initiative, implementation in Africa remains understudied. This scoping review aims to assess the current state of implementing WHO GLASS recommendations on antimicrobial sensitivity testing (AST) in Africa.

**Objective:**

The primary objective of this study is to determine the current state of implementing the WHO GLASS recommendations on AST for priority pathogen-antimicrobial combinations. The review will further document if the reporting of AST results is according to “susceptible,” “intermediate,” and “resistant” recommendations according to GLASS.

**Methods:**

Following the methodological framework by Arksey and O’Malley, studies published between January 2016 and November 2023 will be included. Search strategies will target electronic databases, including MEDLINE, Scopus, CINAHL, and Embase. Eligible studies will document isolates tested for antimicrobial sensitivity, focusing on WHO-priority specimens and pathogens. Data extraction will focus on key study characteristics, study context, population, and adherence to WHO GLASS recommendations on AST. Descriptive statistics involving summarizing the quantitative data extracted through measures of central tendency and variation will be used. Covidence and Microsoft Excel software will be used. This study will systematically identify, collate, and analyze relevant studies and data sources based on clear inclusion criteria to provide a clear picture of the progress achieved in the implementation of the WHO GLASS recommendations. Areas for further improvement will be documented to inform future efforts to strengthen GLASS implementation for enhanced AMR surveillance in Africa.

**Results:**

The study results are expected in August 2024.

**Conclusions:**

To our knowledge, this scoping review will be the first to comprehensively examine the implementation of WHO GLASS recommendations in Africa, shedding light on the challenges and successes of AMR surveillance in the region. Addressing these issues aims to contribute to global efforts to combat AMR.

**International Registered Report Identifier (IRRID):**

PRR1-10.2196/58140

## Introduction

Drug-resistant infections are a leading cause of morbidity and mortality, with 1.5 million related deaths reported in 2019 [1]. Low- and middle-income countries, such as those in Africa, disproportionately face challenges associated with communicable diseases, yet there is limited data on the epidemiology, impact, and geographical distribution of antimicrobial resistance (AMR) in these regions [2] as comprehensive population-based AMR surveillance poses considerable challenges [3-6]. The 2014 World Health Organization (WHO) report on global surveillance of AMR emphasized the lack of information regarding pathogens that pose significant public health risks [7]. Assessing and monitoring AMR trends globally is limited by a lack of quality data [8].

AMR surveillance systems are fundamental elements of infectious disease management [6] and provide the basis for a deeper comprehension of AMR spread [9]. AMR surveillance data can enhance public health, guide health policy decisions, prompt responses to health crises, offer early alerts about emerging threats, and identify longstanding resistance patterns [6]. In low- and middle-income countries, the capability for surveillance of AMR varies [9], with sub-Saharan Africa and Southeast Asia exhibiting the least developed coverage compared with high-income countries [10-12]. In these low-income areas, such as Africa, AMR surveillance challenges are significant due to weak infrastructure, including the underdeveloped treatment infrastructure, lack of adequately trained human resources and limited funding within health systems, various legal and policy limitations, and other socioeconomic factors (culture and societal factors) [13,14]. Despite the above challenges faced, a number of countries in Africa are implementing the WHO Global Antimicrobial Resistance Surveillance System (GLASS) recommendations on AMR surveillance [15]. This is in addition to other tools like the Global Action Plan and the WHO benchmarks [16].

AMR surveillance is 1 of the 5 strategic objectives of the AMR Global Action Plan [3]. To inform surveillance, the WHO launched the GLASS manual in 2015 [15] to standardize surveillance, including antimicrobial sensitivity testing (AST). The WHO GLASS manual makes several recommendations on AMR surveillance systems. Among these include recommendations on priority specimens and pathogens for AMR surveillance of routine clinical samples [15]. In addition, the WHO has identified priority antibiotics for which monitoring of resistance should be done (Table 1) [15]. To achieve this, the GLASS manual recommends pathogen-antimicrobial combinations on which GLASS gathers data [15].

The antimicrobial substances against which resistance or nonsusceptibility will be monitored were selected because either they are commonly recommended first-line treatment or surrogate substances for resistance in drugs commonly used to treat patients, or the pathogen-antimicrobial combination is of particular concern because of limited treatment options. The recommended priority specimens include blood, urine, feces, and urethral and cervical swabs (Table 2) [15]. The priority specimens were selected because they can be used to isolate pathogens that cause most of the common human infections in the bloodstream, urinary tract, gastrointestinal tract, and genital or reproductive tract. The purpose of using priority specimens and pathogens was to enable systematic capacity building for countries as they set up their national AMR surveillance programs [17]. Despite these recommendations, countries could also report on additional specimens and pathogens if national capacity for surveillance of these organisms exists.

Although the GLASS manual was rolled out in 2016, the extent of its implementation, specifically for priority specimens and pathogen-antibiotic combinations, has not been fully explored, especially in Africa. We aim to review existing literature to fill this knowledge gap and inform current and future practices of AMR surveillance. Specifically, we shall review, summarize, and report on priority specimens, pathogen-antibiotic combinations testing, and reporting in the context of the WHO GLASS manual.

**Table 1:** Pathogen-antimicrobial combinations on which the Global Antimicrobial Resistance Surveillance System gathers data.

Pathogen	Antibacterial class	Antibacterial agents that may be used for AST^a,b,c^
*Escherichia coli*	Sulphonamides and trimethoprim Fluoroquinolones Third-generation cephalosporins Fourth-generation cephalosporins Carbapenems Polymyxins Penicillins	Cotrimoxazole Ciprofloxacin or levofloxacin Ceftriaxone or cefotaxime and ceftazidime Cefepime Imipenem, meropenem, ertapenem, or doripenem Colistin Ampicillin
*Klebsiella pneumoniae*	Sulphonamides and trimethoprim Fluoroquinolones Third-generation cephalosporins Fourth-generation cephalosporins Carbapenems Polymyxins	Cotrimoxazole Ciprofloxacin or levofloxacin Ceftriaxone or cefotaxime and ceftazidime Cefepime Imipenem, meropenem, ertapenem, or doripenem Colistin
*Acinetobacter baumannii*	Tetracyclines Aminoglycosides Carbapenems^d^ Polymyxins	Tigecycline or minocycline Gentamicin and amikacin Imipenem, meropenem, or doripenem Colistin
*Staphylococcus aureus*	Penicillinase-stable beta-lactams	Cefoxitin^e^
*Streptococcus pneumoniae*	Penicillins Sulphonamides and trimethoprim Third-generation cephalosporins	Oxacillin^f^ Penicillin G Cotrimoxazole Ceftriaxone or cefotaxime
*Salmonella* spp.	Fluoroquinolones Third-generation cephalosporins Carbapenemsd	Ciprofloxacin or levofloxacin Ceftriaxone or cefotaxime and ceftazidime Imipenem, meropenem, ertapenem, or doripenem
*Shigella* spp.	Fluoroquinolones Third-generation cephalosporins Macrolides	Ciprofloxacin or levofloxacin Ceftriaxone or cefotaxime and ceftazidime Azithromycin
*Neisseria gonorrhoeae*	Third-generation cephalosporins Macrolides Aminocyclitols Fluoroquinolones Aminoglycosides	Cefixime Ceftriaxone Azithromycin Spectinomycin Ciprofloxacin Gentamicin

^a^AST: antimicrobial sensitivity testing

^b^The listed substances are priorities for surveillance of resistance in each pathogen, although they may not be first-line options for treatment. One or more of the drugs listed may be tested.

^c^One or more of the drugs listed may be tested in countries. Susceptible, intermediate, resistant, and nominator and denominator data for each shall be reported separately.

^d^Imipenem or meropenem is preferred to represent the group when available.

^e^Cefoxitin is a surrogate for testing susceptibility to oxacillin (methicillin and nafcillin); the antimicrobial sensitivity testing report to clinicians should state susceptibility or resistance to oxacillin.

^f^Oxacillin is a surrogate for testing reduced susceptibility or resistance to penicillin; the antimicrobial sensitivity testing report to clinicians should state reduced susceptibility or resistance to penicillin.

**Table 2:** Global Antimicrobial Resistance Surveillance System priority specimens and pathogens for the scoping review.

Priority specimens	Priority pathogens
Bloodstream infections	*Klebsiella pneumoniae* *Acinetobacter baumannii* *Staphylococcus aureus* *Streptococcus pneumoniae* *Salmonella spp.*
Urinary tract infections	*Escherichia coli* *Klebsiella pneumoniae.*
Acute diarrhea	*Salmonella spp.* *Shigella spp.*

## Methods

### Study Eligibility

The primary objective of this study is to determine the current state of implementing the WHO GLASS recommendations on AST for priority pathogen-antimicrobial combinations. We shall use a scoping review to map concepts around the study topic. The methodological framework proposed by Arksey and O’Malley [18] (6-step scoping review process) will be adopted for this proposed review [18]. The PRISMA-ScR (Preferred Reporting Items for Systematic Reviews and Meta-Analyses) guidance for scoping reviews will be used to report the study findings [19]. We anticipate that the methodologies of the studies will be more diverse, including observational studies, randomized controlled trials, cohort, and mixed methods studies. We will include only published and peer-reviewed articles (Textbox 1).

**Inclusion and exclusion criteria for the different articles to include in the scoping review.**

**Inclusion criteria**

- The inclusion criteria will be based on the Population, Concept, and Context framework as proposed by the Joanna Briggs Institute for scoping reviews as a less restricted alternative to the Population, Intervention, Comparator, and Outcome framework [20]. Original research articles and short reports with primary data will be included.

**Exclusion criteria**

- Editorials, commentaries, review papers, or any other publication without primary data
- Studies with insufficient data regarding the study question
- Studies not published in English
- Studies published before 2016

### Study Population

The scoping review will include studies that document isolates collected and tested for antimicrobial sensitivity in Africa. Only isolates from the WHO priority specimens will be included. All reported isolates will be assessed to determine whether the WHO priority pathogen-antibiotic combinations were tested as per the WHO GLASS manual recommendations. Our secondary objective for this review will be to document whether reporting of AST results is according to “susceptible,” “intermediate,” and “resistant” recommendations.

### Search Strategy

Search strategies will target the following electronic databases: MEDLINE, Scopus, CINAHL, and Embase. Keywords used for the search will include “Antimicrobial Resistance,” “Anti-microbial Susceptibility,” “AST,” “AMR Surveillance,” “Diagnostic,” “Africa,” and specific names of all African countries (Table 3). Finally, screening of reference lists of included documents for relevant articles will be undertaken. Endnote (Clarivate) will be used to manage citations, Covidence will be used to screen abstracts and full texts, and Microsoft Excel will be used for data extraction and charting the H stages of this review.

**Table 3:** Sample search terms or concepts that will be searched as keywords and subject headings.

Subject headings	Keywords
MeSH^a^ search terms	“Drug resistance” OR “Antimicrobial resistance” OR “Bacterial resistance” OR “Drug Resistance, Bacterial”, OR “Microbial Sensitivity Tests OR “Drug Resistance, Microbial” AND
Text word search	“antibiotic resistan*” OR “antibacterial resistan*” OR “antimicrobial resistan*” OR “antimicrobial drug resistan*” OR “antibiotic drug resistan*” OR “antibacterial drug resistan*” OR “bacteraemia” OR “bacteremia” OR “bloodstream inf*” AND
Title and abstract	“angola” OR “benin” OR “botswana” OR burkina faso” OR “burundi” OR “cameroon” OR “cape verde” OR “central african republic” OR “chad” OR “ivory coast” OR “cote d ivoire” OR “congo” OR “comoros” OR “djibouti” OR “Equatorial Guinea” OR “eritrea” OR “ethiopia” OR “gabon” OR “gambia” OR “ghana” OR “guinea” OR “guinea bissau” OR “kenya” OR “leshoto” OR liberia” OR “madagascar” OR “malawi” OR “Mali” OR “mauritania” OR “mozambique” OR “namibia” OR “niger” OR “nigeria” OR “rhodesia” OR “rwanda” OR “sao tome” OR “senegal” OR “seychelles” OR “sierra leone” OR “somalia” OR “south africa” OR “sudan” “swaziland” OR “tanzania” OR “togo” OR “uganda” OR “zambia” OR “zimbabwe” OR “africa”

^a^MeSH: medical subject headings.

### Data Extraction, Charting, Synthesis, Analysis, and Presentation of Results

#### Data Extraction

After searching the primary sources, articles will be exported from Endnote into Covidence for further screening and analysis. Covidence will be used to remove duplicate sources from the initial pool and complete screening at 2 levels. We shall develop a template for data extraction. Key variables to be extracted will include author and year of publication, study title, inclusion criteria, study type, study setting, study population, priority specimen, priority pathogen, pathogens isolated, antimicrobial agent and class used, and reporting in the context of the WHO GLASS manual and significant findings.

The adapted extraction tool will be used in both the initial stages of study screening (to confirm study relevance) and selection and the later phase of data extraction from the selected studies. The customized data extraction tool will be used to collect relevant information on the (1) key study characteristics (eg, publication year, publication type, study design, country, and patient population characteristics), (2) detailed information on the study context and population, and (3) AST of bacterial isolates against the WHO GLASS recommendations.

To ensure systematic and reproducible study selection and data charting processes and to foster high interrater reliability, a calibration exercise will be undertaken. First, the review lead will use a seminal article to ascertain if the extraction instrument is appropriate for its intended use. Once confidence in the tool has been internally established, 2 members of the review team will be involved in the pilot of the extraction tool, using a minimum of 20 abstracts to review titles and abstracts against the inclusion criteria mentioned earlier. We will review the results of the calibration, discuss any discrepancies among reviewers, and make refinements to the extraction tool as identified and required. Reviewers will, at the same time, document reasons for exclusion on the extraction form and progress those articles considered relevant and eligible to the second phase of full-text screening. Confirmed sources for inclusion in the scoping review will then be moved to the final stages of data extraction, charting, and synthesis.

Following the identification and selection of the relevant literature, we will explore within those studies the AST of isolates according to WHO GLASS pathogen-antibiotic combinations. The reviewers will independently chart data in duplicate from each eligible article. Should there be any disagreements among the reviewers, these will be resolved through discussion. The supervising reviewer will resolve any conflicts and provide oversight for the whole process. We aim to provide a descriptive summary of the gaps in the implementation of the WHO GLASS manual, specifically to AST, based on the priority pathogen-antibiotic recommendation. Where relevant, we intend to suggest how the barriers can be addressed to improve AMR surveillance.

### Primary Data Synthesis and Analysis

Descriptive statistics involving the analysis and summarizing of the data through measures of variability and central tendency will be used. Where required, stratification by factors like study type (cohort, cross-sectional, trails, and others), study settings (community or community), and others will be undertaken at the analysis stage. This will allow for a more nuanced understanding of the implementation progress and help minimize bias at the analysis stage of the review.

### Data Presentation and Dissemination Plan

The data will be provided in well-organized tables and visuals, such as charts, summarizing the quantitative data generated. The data will be published in an international peer-reviewed journal to reach a broad academic and research audience or community. The results will also be presented at conferences.

### Risk of Bias Assessment or Quality Appraisal

Consistent with the Joanna Briggs Institute scoping review methodology, as this is a scoping review that aims to map all available knowledge regarding the study topic, we will not perform a risk of bias assessment. We shall appraise articles for quality using the Critical Appraisal Skills Programme (CASP) checklist. Where articles of low quality are included, a comment will be made.

### Patient and Public Involvement

Patients will not be directly involved in this study. However, the findings will be published for the wider community and policymakers, with the view that they could improve public health and contribute to efforts to control AMR.

### Ethical Considerations

No ethical clearance is needed for this study as it will be based on already published articles, and no human participants will be involved directly in the study.

## Results

The results of the study are expected in August 2024.

## Discussion

### Expected Findings

This proposed review is intended to map evidence of the progress in implementing the WHO GLASS recommendations on AST for priority pathogen-antimicrobial combinations [15]. It will inform current and future practices of AMR surveillance, provide ground for further research on GLASS implementation activity research, and inform policy makers on areas of improvement in AMR control activities. Through conducting surveillance of specific pathogens over time, one can detect emerging pathogens and implement timely control, understand epidemiological trends, discover changes in the antimicrobial susceptibility profile of the organisms, monitor and inform treatment guidelines, and facilitate outbreak response [21,22].

Despite the great outcomes and strengths we anticipate from this study, several limitations are still envisaged. The fact that the review is to be conducted with reference to the first WHO GLASS manual 2015, a small scope for pathogens and priority pathogen-antibiotic combinations, will be considered.

### Conclusions

AMR surveillance is one of the critical approaches to estimate and fight the burden of antibiotic resistance. This scoping review will be the first to provide information on the progress of implementation of the WHO GLASS initiative in Africa.

## Abbreviations

AMR: antimicrobial resistance
AST: antimicrobial sensitivity testing
CASP: Critical Appraisal Skills Programme
GLASS: Global Antimicrobial Resistance Surveillance System
PRISMA-ScR: Preferred Reporting Items for Systematic Reviews and Meta-Analyses) guidance for scoping reviews
WHO: World Health Organization

## Data Availability

All data generated or analyzed during this study will be included in the published paper and its supplementary information files.
